# Detection of border disease virus (BDV) in goat herds suffering diarrhea in eastern China

**DOI:** 10.1186/1743-422X-10-80

**Published:** 2013-03-11

**Authors:** Wenliang Li, Li Mao, Yongqian Zhao, Yinhua Sun, Kongwang He, Jieyuan Jiang

**Affiliations:** 1 Institute of Veterinary Medicine, Jiangsu Academy of Agricultural Sciences; Key Laboratory of Veterinary Biological Engineering and Technology, Ministry of Agriculture; National Center for Engineering Research of Veterinary Bio-products, Nanjing, 210014, China; 2 Hai’an animal husbandry and veterinary station, Hai’an, 226600, China

**Keywords:** Border disease virus, Goat, Diarrhea, Phylogenetic analysis, Eastern China

## Abstract

**Background:**

Border disease virus (BDV) is an important pathogen in sheep and goat production. Neither epidemiological investigation nor any reports of BDV infection was available in China. During Jan to Apr, 2012, several herd goats in Anhui and Jiangsu provinces in eastern China suffered unremitting diarrhea, with morbidity and mortality of about 28-37% and 10-15%, respectively. In the present study, sera and tissue samples from diseased goats of four farms were taken for BDV detection, isolation and identification.

**Results:**

Panpesti generic primers and border disease virus (BDV)-specific primers targeting the 5’-UTR region produced RT-PCR positive bands for sera (24/28) and tissue samples (7/30). Twenty positive sera and tissue samples were inoculated onto Madin-Darby bovine kidney (MDBK) cells for virus isolation. Finally, three different strains of BDV, named AH12-01, AH12-02 and JS12/04, were successfully isolated as identified by RT-PCR using 5’-UTR and N^pro^ gene primers, sequencing and electron microscopy. Sequences of 5’-UTR and N^pro^ genes of them were used for phylogenetic analysis and comparison to other reference sequences available in GenBank. The results indicated AH12-01, AH12-02 and JS12/04 possess high relationship with the BDV 3 group viruses and differed with each other.

**Conclusion:**

This is the first detection of BDV from goats with diarrhea and confirmation of BDV infection in China.

## Background

Border disease virus (BDV) causes a congenital viral disease of sheep and goats. It was first reported at the border of Wales and England, and is characterized by reproductive manifestations such as barren ewes, abortion, stillbirth and the birth of small, weak lambs that manifest tremor, abnormal body conformation and hairy fleeces [1]. BDV belongs to the genus *Pestivirus* of the family *Flaviviridae*, together with bovine virus diarrhea viruses 1 and 2 (BVDV 1, 2) and classical swine fever virus (CSFV) [2,3]. In some reported cases, BDV also caused mucosal disease-like lesions in sheep [4,5].

Border disease is usually transmitted by horizontal and vertical routes, and weak lambs can be persistently infected (PI) [6]. Distribution of BDV is worldwide and the prevalence rate varies between countries and regions. Several studies have confirmed the prevalence of BDV in several countries [6-11]. However, to date, no epidemiological survey has been done in small ruminants (sheep, goat) and there have been no reports of BDV in China.

In this study, BDV was detected by RT-PCR from the serum and tissue samples of diseased goats suffering diarrhea. Virus isolation was performed and confirmed by RT-PCR and sequencing. Three different strains were isolated and phylogenetic analysis was performed based on 5’-UTR and N^pro^ sequences. This is the first report confirming the occurrence of BDV infection in Chinese goat herds, which might contribute to the clinical disease.

## Results

### Clinical observations

**Figure 1:**
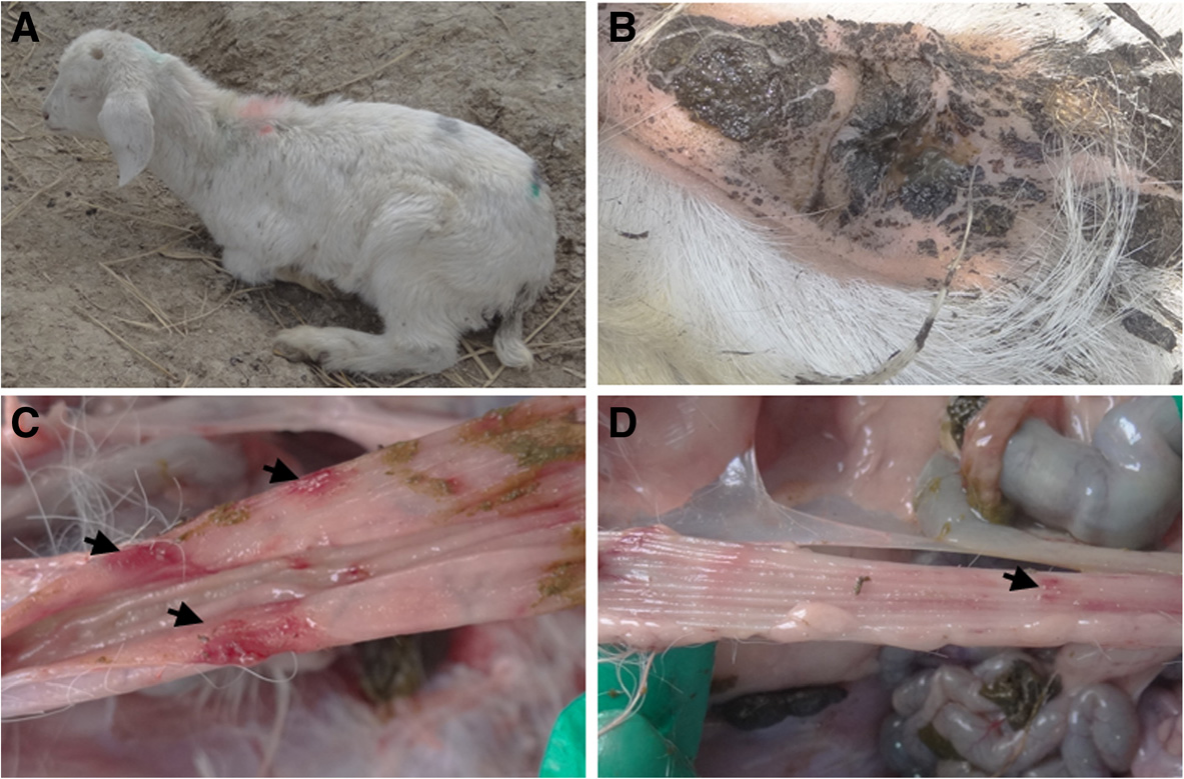
**Clinical and pathological observations of diseased goats. A**. diseased goat showed depression, rough hair-coat and growth retardation; **B**. goat with diarrhea. The anus and neighboring fur were covered with loose stool; **C** and **D**. hemorrhage and necrosis in small and large intestine (black arrows).

The clinical signs included serious and repeated diarrhea, rough hair-coat, depression, and slow growth (Figure 1A, B). Necropsy of the diseased goats (2–4 months of age) showed gross pathological lesions in the alimentary tract. Obvious hemorrhage was observed in the stomach, small intestine and large intestine (Figure 1C, D). 4 out of 5 necropsied goats showed slightly or moderate enlarged mesenteric lymph nodes, while no obvious lesions were observed in other organs including lung, heart, liver, spleen, kidney and brain. Gastrointestinal parasites were not found.

### RT-PCR detection and virus isolation

**Figure 2:**
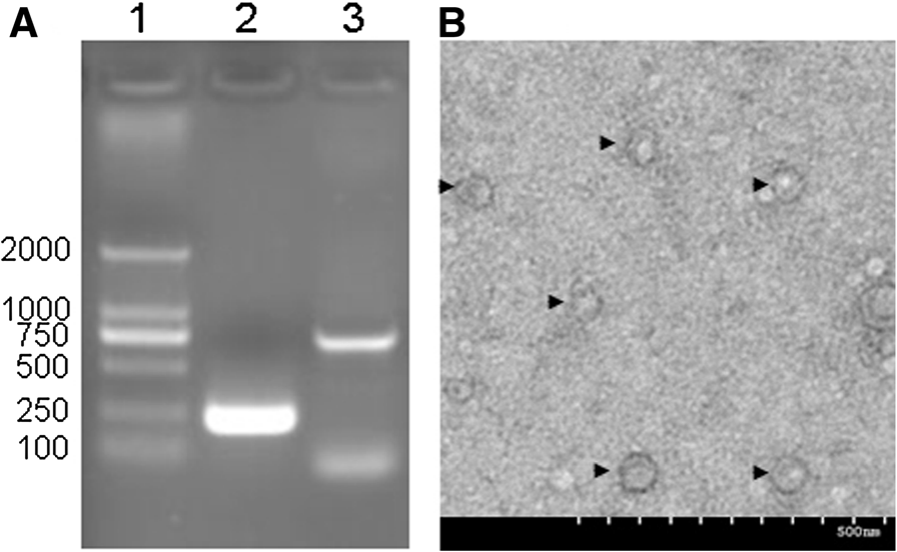
**RT-PCR (A) and electron microscopy (B) detection of isolated BDV virus(only the result of the third passage of AH12-01 was shown). A**. lane 1: DNA molecular weight standard DM 2000; lane 2: 5’-UTR RT-PCR products (225bp); lane 3: N^pro^ RT-PCR products (736bp); **B**. several 50–60 nm viral particles were observed by electron microscopy (black arrows); each graduate in the scale bar represents 50 nm.

As for RT-PCR detection, nearly all sera samples (24/28) and some of the tissue samples (7/30, spleen and lymph nodes, from 4 goats) gave positive results (Table 1). Virus isolation was performed on MDBK cells and 3 serial passages were performed. No CPE was observed. Cell cultures of the first and third passages were examined by RT-PCR and clear bands could be seen (Figure 2A). A total of 16 sera and 4 tissue samples were used for virus isolation. Based on the sequence analysis, three different strains were obtained finally and were named as AH12-01, AH12-02 and JS12/04. AH12-01 was isolated from farm A and C; AH12-02 was isolated from farm A and B; JS12/04 was isolated from farm D (Table 1). The nucleotide sequences of 5'-UTR-N^pro^ regions (949 or 952bp) have been deposited in GenBank (JQ946320, AH12-01; KC537788, AH12-02; KC537789, JS12/04). In addition, numerous viral particles with a size of 50-60nm in diameter were observed by electron microscopy (Figure 2B). These results suggested the success isolation of BDV.

**Table 1:** **Summary of the sampling**, **RT**-**PCR detection and virus isolation results**

**Farm**	**Location**	**Size**	**Sera samples Positive/total**	**Tissue samples Positive/total**	**Virus isolation**
A	Anhui	160	7/8	3/12	AH12-01 AH12-02
B	Jiangsu	180	5/7	2/12	AH12-02
C	Jiangsu	150	6/6	2/6	AH12-01
D	Jiangsu	200	6/7	0/0	JS12/04

### Phylogenetic analysis

**Figure 3:**
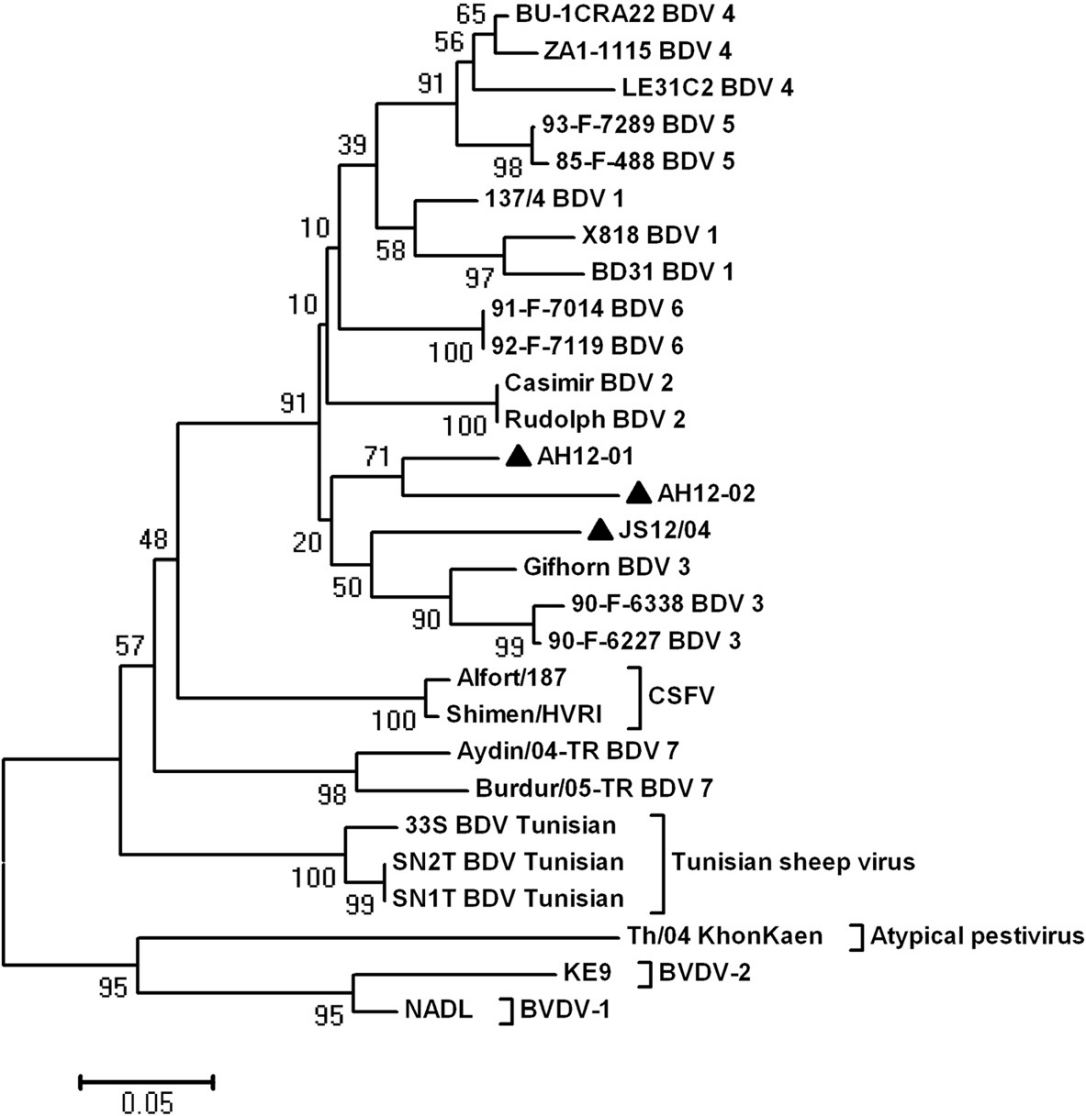
**Neighbor-joining unrooted trees based on the 5'-UTR sequences.** Numbers at nodes indicate bootstrap percentages obtained after 1,000 replicates. Scale bar indicates genetic distance. Our BDV isolates were marked with ▲. The sequences used in the phylogenetic analysis are as follows: U70263, BD31; AF037405, X818; U65052, 137/4; DQ361072, LE31C2; AB122085, Casimir; AB122086, Rudolph; GQ902940, Gifhorn; EF693991, 90-F-6338; EF693989, 90-F-6227; DQ275622, BU-1CRA22; DQ361070, ZA1-1115; EF693995, 93-F-7289; EF693985, 85-F-488; EF693993, 91-F-7014; EF693994, 92-F-7119; AM418427, BDV/Aydin/04-TR; AM418428, BDV/Burdur/05-TR; AF461997, SN1T; AF461996, SN2T; AF462002, 33S; NC_012812, Th/04_KhonKaen; M31182, NADL; EF101530, KE9; AY775178, Shimen/HVRI; X87939, Alfort/187.

**Figure 4:**
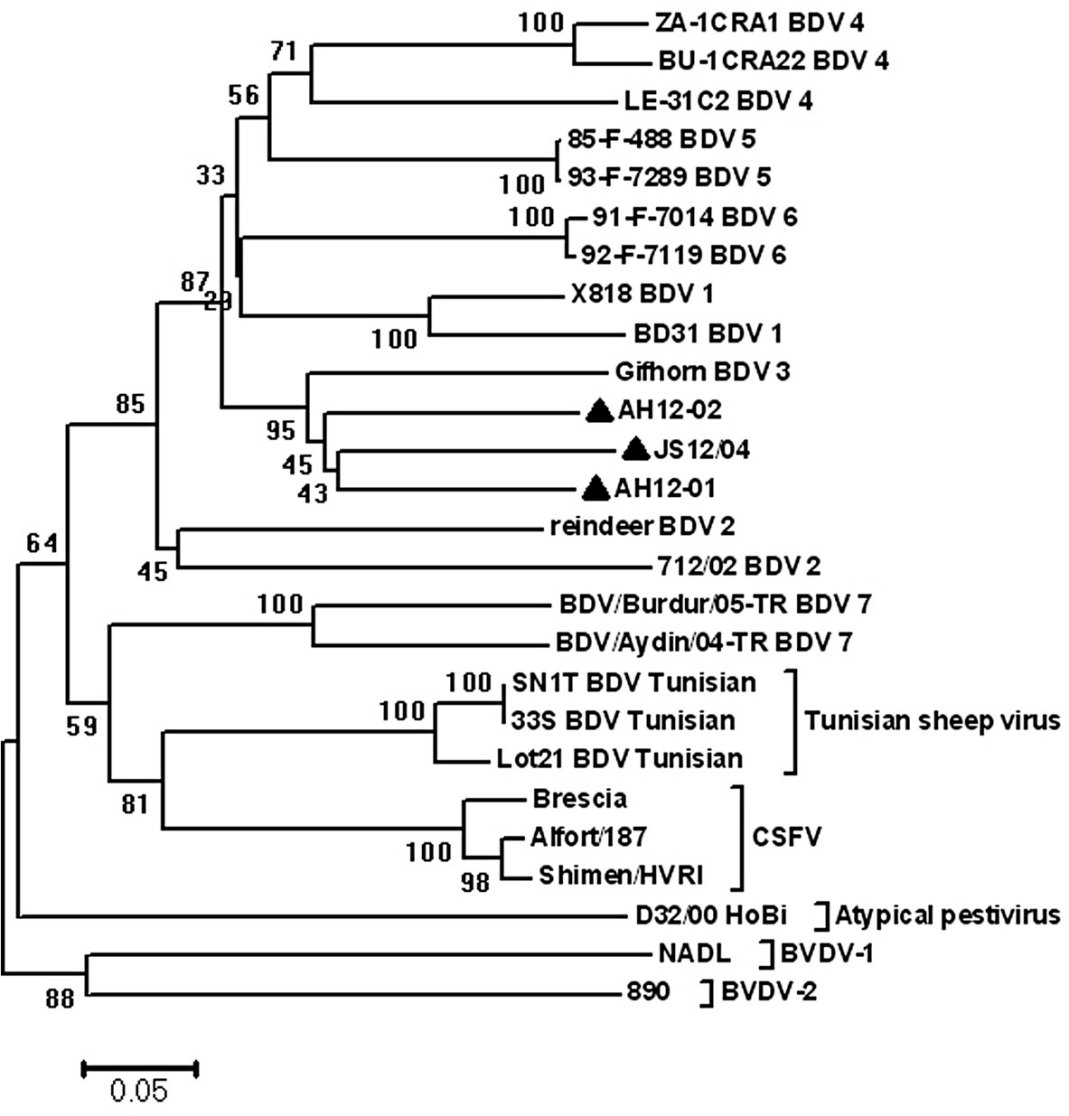
**Neighbor-joining unrooted trees based on the N**^**pro **^**gene sequences.** Numbers at nodes indicate bootstrap percentages obtained after 1,000 replicates. Scale bar indicates genetic distance. Our BDV isolates were marked with ▲. The sequences used in the phylogenetic analysis are as follows: U70263, BD31; AF037405, X818; AF144618, reindeer; AJ829444, 712/02; AY163653, Gifhorn; DQ273161, LE-31C2; DQ273155, BU-1CRA22; DQ273164, ZA-1CRA1; EF693963, 85-F-488; EF693973, 93-F-7289; EF693971, 91-F-7014; EF693972, 92-F-7119; EU930014, BDV/Aydin/04-TR; EU930015, BDV/Burdur/05-TR; AF462010, Lot21; AY452484, SN1T; AY452485, 33S; AY735486, D32/00_HoBi; M31182, NADL; U18059, 890; AY775178, Shimen/HVRI; X87939, Alfort/187; AF091661, Brescia.

The phylogenetic analysis of 5’-UTR sequences showed that AH12-01, AH12-02 and JS12/04 were closely grouped with the reference Gifhorn strain of BDV3 (Figure 3). These strains possessed nucleotide identity of 87.7%-91.5% with each other and they shared 75.7%-90.6% nucleotide identity with other BDV strains (Table 2). The N^pro^ sequences based phylogenetic analysis showed a similar result, with higher bootstrap values (Figure 4). AH12-01, AH12-02 and JS12/04 showed lower nucleotide diversity with each other and other BDV strains (Table 2). These results suggest that these three isolates might belong to BDV 3 group.

**Table 2:** **5**’-**UTR and N**^**pro**^**nucleotide identity of isolated strains with other BDVs** (**BDV1**-**7**)

**5’-UTR**	**Type**	**AH12-01**	**AH12-02**	**JS12/04**	**N**^**pro**^	**Type**	**AH12-01**	**AH12-02**	**JS12/04**
AH12-01		-	91.5	90.2	AH12-01		-	81.7	81.5
AH12-02		91.5	-	87.7	AH12-02		81.7	-	81.5
JS12/04		90.2	87.7	-	JS12/04		81.5	81.5	-
Gifhorn	BDV3	90.2	86	89.4	Gifhorn	BDV3	79.1	79.9	79.5
90-F-6227	BDV3	89.4	87.2	90.6	X818	BDV1	77.2	74.1	73.9
90-F-6338	BDV3	89.8	88.1	89.8	BD31	BDV1	74.9	71.9	73.5
X818	BDV1	87.2	86.8	85.5	reindeer	BDV2	74.1	75.4	73.3
BD31	BDV1	84.3	84.7	81.7	712/02	BDV2	71.5	69.4	71
137/4	BDV1	89.8	86.4	87.7	ZA-1CRA1	BDV4	75.2	71.7	73.5
Casimir	BDV2	89.4	85.5	86.4	BU-1CRA22	BDV4	73.9	71.7	72.3
Rudolph	BDV2	89.4	85.5	86.4	LE-31C2	BDV4	74.1	74.9	74.5
ZA-1CRA1	BDV4	87.2	85.1	86.8	85-F-488	BDV5	74.5	75.8	74.5
BU-1CRA22	BDV4	88.9	86.8	88.1	93-F-7289	BDV5	74.7	75.6	74.7
LE-31C2	BDV4	86.4	86.4	85.5	91-F-7014	BDV6	76.2	76.8	72.9
85-F-488	BDV5	87.7	85.5	87.2	92-F-7119	BDV6	76	77.4	73.1
93-F-7289	BDV5	88.5	85.1	87.2	Burdur/05-TR	BDV7	65.1	67.1	67.4
91-F-7014	BDV6	87.7	84.3	86.4	Aydin/04-TR	BDV7	65.9	66.4	67.1
92-F-7119	BDV6	87.7	84.3	86.4					
Burdur/05-TR	BDV7	78.3	79.1	75.7					
Aydin/04-TR	BDV7	79.1	80.4	77					

## Discussion

Distribution of BDV is worldwide and the prevalence has been confirmed in several countries. Most reports have studied BDV in sheep, cattle or *Pyrenean chamois*[6], rarely in goat . In addition, no epidemiological survey has been reported in small ruminants in China. In this study, three different BDV strains were detected from goat herds suffering intractable diarrhea.

Both BVDV and BDV infect cattle, sheep, goats, and many other wild ruminants and pigs. But in clinical conditions, BVDV is mostly found in cattle, whereas both BDV and BVDV can be isolated from sheep [12]. In this study, BVDV infection was firstly considered and detected by using RT-PCR with the panpesti generic primers. Sequencing the RT-PCR products resulted in the detection of BDV, as further confirmed with BDV isolation. Infections of pestivirus can vary from subclinical to clinical signs such as fever, diarrhea, hemorrhagic syndrome, death and abortion. No classical symptoms (abortion, stillbirth and the birth of small, weak lambs) were observed in tested goat herds; only diarrhea and alimentary tract lesions were the major clinical and pathologic signs. Barlow et al., Monies et al. and Chalmers et al. have reported that BDV caused mucosal disease-like lesions in sheep, which was characterized by unremitting diarrhea [4,5,13]. Few studies have done for BDV infection in goat herds [14-16]; and no diarrhea related report was available. In these cases, the development of clinical disease might be multifactoral. Detection of other diarrhea related viruses and bacteriological examinations have not been performed yet. So BDV infection might be one of the pathogenic factors causing the diarrhea of the goats here. In order to determine the pathogenicity of BDV isolates on goats, especially for diarrhea, animal infection experiments should be done.

The genetic diversity of BDV is greater than that reported for other pestivirus species. BDV have been divided into six groups: BDV 1–6 [10]. Several isolates from Tunisian [17] and Turkey [1] represented new groups. Based on sequence alignment and phylogenetic analysis, the strains isolated in this study showed 75.7%-90.6% and 65.1%-79.9% nucleotide identities with other 5’-UTR and N^pro^ sequences (BDV1-7), indicating the high diversity of different BDV genotypes and the potential variation of Chinese BDV strains.

BDV infection has not yet been reported in China, and no study has been performed on the epidemiology of BDV. In this study, BDV was detected from different farms in different region and the virus infection was confirmed by RT-PCR, virus isolation and electron microscopy. Three strains were isolated from four farms. AH12-01 and AH12-02 were detected in two farms; and farm A was infected with both AH12-01 and AH12-02 strains (Table 1), suggesting the complicated epidemic situation. At present the origin of the virus is unknown. It may have existed in the Chinese goat herd for some time without being noticed or other possibilities of virus introduction. All these results highlight the need for serological assay development to explore the prevalence of BDV in China both in etiologic and serological levels. More samples from different herds in Jiangsu, Anhui provinces as well as more provinces of China are under collection in our lab for epidemiological survey and we hope the results may help us understand the origin and prevalence status of BDV in China.

## Conclusions

BDV infection was confirmed in goat herds suffering intractable diarrhea by virus isolation, sequencing and phylogenetic analysis. This is the first report of BDV prevalence in Chinese goat herds.

## Methods

### Cases and sampling

From Jan to Apr 2012, goats of four farms in Anhui and Jiangsu provinces (one in Anhui (farm A), three in Jiangsu (farm B, C, D)) in eastern China suffered severe and unremitting diarrhea, with morbidity and mortality of about 28-37% and 10-15%, respectively. These four goat herds were fattening farms, which purchase weaning goats from several small backyard farms for fatten until slaughter time. Herds size ranged between 150 and 200 animals. Diarrhea was observed for previous batches, but was not such severe and most of the diseased goats recovered after treatment with antibiotics. The goats were vaccinated against goatpox virus, *Clostridium septicum*, *Clostridium welchii* and *Clostridium novyi*. In this present case, several kinds of antibiotics were used, but did not have any positive effect. Twenty eight sera samples from 28 diseased goats (farm A: 8, B: 7, C: 6, D: 7) and 30 tissue samples (brain, liver, lung, spleen, kidney and mesenteric lymph nodes) from 5 necropsied goats (farm A: 2, B: 2, C: 1) were taken and stored at −70°C for RT-PCR and virus isolation (Table 1).

### Ethical approval

The collection of sera and tissues samples was performed in strict accordance with the guidelines of Jiangsu Province Animal Regulations (Government Decree No 45).

### RT-PCR

Viral RNA was extracted from sera and tissues samples using TRIzol reagent (Invitrogen) according to the manufacturer’s instruction. Reverse transcription was performed for 1 h at 42°C in a mixture including 8 μl total RNA, 1 μl EasyScript RT/RI Enzyme Mix, (Transgen, Bio, Inc.)1 μl reverse primer and 10 μl 2×ES buffer. PCR amplification (Transgen, Bio, Inc.) was carried out in a 50 μl reaction mixture containing 1× PCR buffer, 0.2 mM of dNTPs, 20 pM of each primer (panpesti generic primers and BDV specific primers PBD1/PBD2 targeting 5’-UTR, with expected product sizes of 290bp and 225bp, respectively) [8,18], 2 U of *Taq* DNA polymerase (Transgen, Bio, Inc.) and 4 μl of cDNA. The reaction was run in a thermocycler (Mjmini, BIO-RAD) with the following program: denaturation at 94°C for 5min, 35 cycles composed of denaturation at 94°C for 30 s, annealing at 54°C for 30 s and extension at 72°C for 45s, and was terminated with a final extension of 10 min at 72°C. Amplification products were detected by electrophoresis in 1.2% agarose gels.

### Virus isolation

Positive tissue samples were homogenized in 5 ml of phosphate-buffered saline (PBS, pH 7.2), and then frozen and thawed 3 times. After centrifugation at 12,000 rpm for 20 min, the supernatant was filtered through 0.22 μm filter (Millipore) and inoculated onto Madin-Darby bovine kidney (MDBK) cell monolayers. Positive sera samples were used for inoculation directly after centrifugation at 12,000 rpm for 20 min. The MDBK cultures were observed for 5 days. Cell cultures were harvested and passaged 2 more times. Each viral stock was stored at −70°C for RT-PCR detection. Virus isolation was confirmed by RT-PCR using primers PBD1/PBD2 and primers targeting the N^pro^ gene (320F/1040R, with RT-PCR product of 736bp) [8,12].

### Electron microscopy

MDBK cells were infected with the 3rd viral passage stock. A total of 200 ml virus stock was harvested. Cell debris was removed by low speed centrifugation (8,000×*g* for 0.5 h) and the supernatants were ultracentrifuged at 60,000×*g* for 2 h. The resulting pellet was dissolved in PBS and stained with phosphotungstic acid (PTA), blotted dry, and examined with an electron microscope (H-7650, HITACHI).

### Gene clone, sequencing and phylogenetic analysis

The 290bp 5’-UTR and the 736bp N^pro^ gene fragments of the isolates were amplified, purified with a DNA purification kit (Axygen Bio, Inc.), cloned into pMD18-T vector (Takara Bio, Inc.), and transformed into *E*. *coli* DH5α competent cells. Positive plasmids were confirmed by restriction enzyme analysis and sequencing. Each sequence of the PCR products was determined for both DNA strands by sequencing.

The nucleotide sequences were edited by Editseq (DNASTAR Inc., Madison, WI) to obtain ≈950bp 5’-UTR-N^pro^ sequences by deleting the overlap region. Multiple sequence alignment was done by using Clustal X 1.83 [19] and MegAlign (DNASTAR Inc., Madison, WI), together with other representative sequences. The 225bp 5’-UTR fragments (PBD1/PBD2 product) and 487bp N^pro^ gene (corresponding to 394-880bp of Gifhorn genome) sequences were used for the analysis, respectively. After determining the percentages of sequence identity among different BDV and other pestivirus strains (CSFV, BVDV-1, BVDV-2, and atypical pestivirus), the phylogenetic tree was generated with the distance-based neighbor-joining (NJ) method by using MEGA 4.0.2 software [20]. The robustness of the phylogenetic tree was determined by bootstrap resampling analysis carried out on 1000 replicates.

## Abbreviations

BDV: Border Disease Virus; BVDV: Bovine Viral Diarrhea Virus; CPE: Cytopathic Effects; CSFV: Classical Swine Fever Virus; MDBK: Madin-Darby Bovine Kidney; PCR: Polymerase Chain Reaction; PI: Persistently Infected; 5’-UTR: 5’-untranslated region.

## Competing interests

The authors declare that they have no competing interests.

## Authors’ contributions

WL, LM and JJ participated in the design and conducted the majority of the experiments in the study. WL drafted the manuscript. YS and YZ contributed to the samples collection. LM performed analyses of data. KH and JJ revised the manuscript. All authors read and approved the final manuscript.
